# A comparative survey between cascade correlation neural network (CCNN) and feedforward neural network (FFNN) machine learning models for forecasting suspended sediment concentration

**DOI:** 10.1038/s41598-024-61339-1

**Published:** 2024-05-09

**Authors:** Bhupendra Joshi, Vijay Kumar Singh, Dinesh Kumar Vishwakarma, Mohammad Ali Ghorbani, Sungwon Kim, Shivam Gupta, V. K. Chandola, Jitendra Rajput, Il-Moon Chung, Krishna Kumar Yadav, Ehsan Mirzania, Nadhir Al-Ansari, Mohamed A. Mattar

**Affiliations:** 1 Department of Agricultural Engineering, Institute of Agricultural Sciences, Banaras Hindu University, Varanasi, Uttar Pradesh 221005 India; 2 Department of Soil and Water Conservation Engineering, Acharya Narendra Deva University of Agriculture & Technology, Kumarganj, Ayodhya, Uttar Pradesh 224229 India; 3 Department of Irrigation and Drainage Engineering, Govind Ballabh Pant University of Agriculture and Technology, Pantnagar, Uttarakhand 263145 India; 4 Department of Water Engineering, Faculty of Agriculture, University of Tabriz, Tabriz, 5166616471 Iran; 5 Department of Railroad Construction and Safety Engineering, Dongyang University, 36040 Yeongju, South Korea; 6 Department of Irrigation and Drainage Engineering, Acharya Narendra Deva University of Agriculture & Technology, Kumarganj, Ayodhya, Uttar Pradesh 224229 India; 7 Division of Agricultural Engineering, ICAR-Indian Agricultural Research Institute, New Delhi, 110012 India; 8 Department of Water Resources and River Research, Korea Institute of Civil Engineering and Building Technology, Goyang-si, 10223 Republic of Korea; 9 Faculty of Science and Technology, Madhyanchal Professional University, Ratibad, Bhopal, 462044 India; 10 Environmental and Atmospheric Sciences Research Group, Scientific Research Center, Al-Ayen University, Thi-Qar, Nasiriyah, 64001 Iraq; 11 Department of Civil, Environmental, and Natural Resources Engineering, Lulea University of Technology, 97187 Luleå, Sweden; 12 Department of Agricultural Engineering, College of Food and Agriculture Sciences, King Saud University, P.O. Box 2460, Riyadh, 11451 Saudi Arabia

**Keywords:** Cascade correlation neural network, Feedforward neural network, Suspended sediment concentration, Machine learning, Seonath basin, Environmental sciences, Hydrology, Applied mathematics

## Abstract

Suspended sediment concentration prediction is critical for the design of reservoirs, dams, rivers ecosystems, various operations of aquatic resource structure, environmental safety, and water management. In this study, two different machine models, namely the cascade correlation neural network (CCNN) and feedforward neural network (FFNN) were applied to predict daily-suspended sediment concentration (SSC) at Simga and Jondhara stations in Sheonath basin, India. Daily-suspended sediment concentration and discharge data from 2010 to 2015 were collected and used to develop the model to predict suspended sediment concentration. The developed models were evaluated using statistical indices like Nash and Sutcliffe efficiency coefficient (N_ES_), root mean square error (RMSE), Willmott’s index of agreement (WI), and Legates–McCabe’s index (LM), supplemented by a scatter plot, density plots, histograms and Taylor diagram for graphical representation. The developed model was evaluated and compared with CCNN and FFNN. Nine input combinations were explored using different lag-times for discharge (Q_t-n_) and suspended sediment concentration (S_t-n_) as input variables, with the current suspended sediment concentration as the desired output, to develop CCNN and FFNN models. The CCNN4 model with 4 lagged inputs (S_t-1_, S_t-2_, S_t-3_, S_t-4_) outperformed the other developed models with the lowest RMSE = 95.02 mg/l and the highest N_ES_ = 0.0.662, WI = 0.890 and LM = 0.668 for the Jondhara Station while the same CCNN4 model secure as the best with the lowest RMSE = 53.71 mg/l and the highest N_ES_ = 0.785, WI = 0.936 and LM = 0.788 for the Simga Station. The result shows the CCNN model was better than the FFNN model for predicting daily-suspended sediment at both stations in the Sheonath basin, India. Overall, CCNN showed better forecasting potential for suspended sediment concentration compared to FFNN at both stations, demonstrating their applicability for hydrological forecasting with complex relationships.

## Introduction

Suspended sediment is typically defined as sediment carried by a fluid in such a way that the force of turbulent eddies is stronger than the particles tendency to settle through the fluid^1^. It affects in rivers significantly impact water quality^2,3^. Precise prediction of suspended sediment load in rivers play a crucial role in both environmental science and the development of engineering infrastructure^4^. They are essential for effective watershed management strategies^4,5^. Sediment outflow from the agricultural land due to rainfall and runoff action leads to a reduction in soil fertility^6–9^. Sediment flows using two routes to reach the watershed outlet; the first is through suspension, and second is through rollover along the land surface as bed load^10,11^. Since sedimentation can lead to floods as deposition of sediment in canal/stream/river, reservoir significantly decreases the depth of flow by virtue of rising in bed, and decrease in live storage capacity of reservoir^4^. In addition, sedimentation significantly affects the intakes of turbines for hydropower plants^12^. Thus, accurate estimation of sediment outflow is desired for better planning, designing, and maintaining water resources structures for water supply, irrigation, drainage, flood control, soil and water conservation, and water quality control^13–16^. In line with the requirement for effective tools for the prediction of sediment yield, it is becoming necessary to develop models capable of estimating sediment outflow^17^. Owing to the complex and nonlinearity of sediment models, it has always been challenging to develop model capable of forecasting exact amount of sediment outflow^18,19^.

Many kinds of researches have been conducted for sediment modelling by using traditional mathematical models like sediment rating curve (SRC)^20,21^ and multiple linear regression (MLR)^22,23^, and they concluded that these models were incapable of model sediment yield^22–25^. Different conventional techniques were analysed to estimate discharge and suspended sediment concentration^26–29^. The conventional models are less effective for sediment computation based on the previous researches. In recent years, machine learning (ML) techniques have been used to overcome problems faced when conventional modelling is attempted^30–36^. Among various ML techniques, artificial neural network (ANN) is the most popular for estimating sediment load^37^, and has provided good results compared to the traditional MLR and SRC methods^38–44^.

Rahul et al.^45^ compared feedforward backpropagation neural network (FFBPNN) and support vector machine (SVM) to forecast suspended sediment concentration at the Varanasi cross-section of the Ganga River. The results indicated that, for validation, the FFBPNN (RSME = 176.2, R = 0.955, and N_ES_ = 0.912) exhibited greater precision in predicting suspended sediment load compared to SVM (RSME = 222.1, R = 0.930, and N_ES_ = 0.864). This study highlights the robustness of soft computing techniques for suspended sediment load prediction. The predictive capability of random subspace (RSS) for predicting suspended sediment load in the Haraz River, Iran, was compared with commonly used methods: random forest (RF) and two machine SVM models using radial basis function kernel (SVM-RBF) and normalized polynomial kernel (SVM-NPK)^46^. The results revealed that the RSS model provided superior predictive accuracy (N_ES_ = 0.83) compared to SVM-RBF (N_ES_ = 0.80), SVM-NPK (N_ES_ = 0.78), and RF (N_ES_ = 0.68). Additionally, the RBF kernel showed better performance than the NPK kernel. Rajaee et al.^47^ compared the wavelet based ANN (WANN), ANN, MLR and conventional sediment rating curve and found the performance of WANN better as compared to the ANN, MLR and conventional sediment RC techniques in the Yadkin Riverat Yadkin College, NC station in the USA. Sahoo et al.^48^ compared selective multimodal Long Short-Term Memory network (SM-LSTM) framework with Long Short-Term Memory network (LSTM) and Recurrent Neural Network (RNN) models to forecast daily suspended sediment loads at two monitoring stations, namely Thebes on the Mississippi River and Omaha on the Missouri River. Comparative analysis of prediction accuracies highlighted that the SM-LSTM model significantly outperformed LSTM and RNN, showcasing its better ability to predict daily water level patterns. Sahoo et al.^48^ emphasizes the potential of deep learning in environmental monitoring and management, particularly in predicting sediment dynamics, which is crucial for maintaining water quality and ecosystem health. Studies have shown the effectiveness of models like the radial M5 tree (RM5Tree) model^49^, adaptive neuro-fuzzy models (ANFIS)^50–53^, multilayer perceptron (MLP)^54^, support vector machine (SVM) models^55–59^, and the coupled Soil and Water Assessment Tool (SWAT) with long short-term memory (LSTM) model^60^. These models have demonstrated accurate sediment yield estimations by utilizing hydro-meteorological variables like temperature, rainfall, discharge, and sediment data. The use of these advanced algorithms can provide reliable predictions even in data-scarce situations, as seen in various watershed studies, enhancing watershed management and engineering structure design, as evident from the research findings^61^.

In the current study, the potential of two different machine learning algorithms including cascade correlation neural network (CCNN) and feedforward neural network (FFNN) were investigated for forecasting daily-suspended sediment load in Sheonath basin, India. The CCNN model has potentially used to examine the capability for predicting/forecasting the different hydrological variables. Karunanithi et al.^62^ investigated the potential of CCNN model for discharge prediction at the Dexter station, Huron River. Alok et al.^63^ predicted river flow of Bramani basin, India. Kim et al.^64^ compared CCNN and multilayer perceptron (MLP) models for predicting daily evaporation, South Korea. Ghorbani et al.^65^ applied CCNN and random forest (RF) models to predict daily river flow using stage-discharge at Dulhunty and Herbert stations, Australia. Also, Zounemat-Kermani et al.^66^ examined the prediction of surface water quality parameters (e.g., water temperature, dissolved oxygen (DO), total dissolved solid (TDS), and pH etc.) in the St. Johns River, Florida. Similarly, FFNN model has been widely utilized by different researchers. Bilhan et al.^67^ compared different models (FFNN and RBNN) with the conventional technique for simulating lateral outflow in channel and found FFNN model superior than RBNN (RMSE = 0.037). Kisi^68^ applied WGRNN GRNN and FFNN models for the prediction of monthly streamflow in two different rivers and found WGRNN outperformed than the GRNN and FFNN model (RMSE = 5.31 m^3^/s, and R = 0.728). Zounemat-Kermani et al.^69^ assessed the performance of FFNN model for predicting daily streamflow in Cahaba River, Alabama. Ehteram et al.^70^ investigated FFNN model with an evolutionary algorithm for estimating suspended sediment concentration yield in the Atrek basin, Iran. Heddam et al.^71^ evaluated the potential of different machine learning models to predict phycocyanin pigment of surface water in the river basin.

The objective of this study is to investigate the use of machine learning models, specifically CCNN and FFNN, for forecasting daily-suspended sediment concentration in the Sheonath basin, India, with a focus on short time-series data. The study also compares the performance of these models and evaluates their suitability for practical application in hydrological organizations within the Sheonath basin. The novelty of current research work is to develop a suspended sediment concentration model based on short time-series data. A comparison of the CCNN models and the FFNN models was also made with the data generated by the corresponding CCNN and FFNN models, and the results were compared in the end. Its performance is assessed statistically and compared with observed data.

## Methodology

### Study area

**Figure 1:**
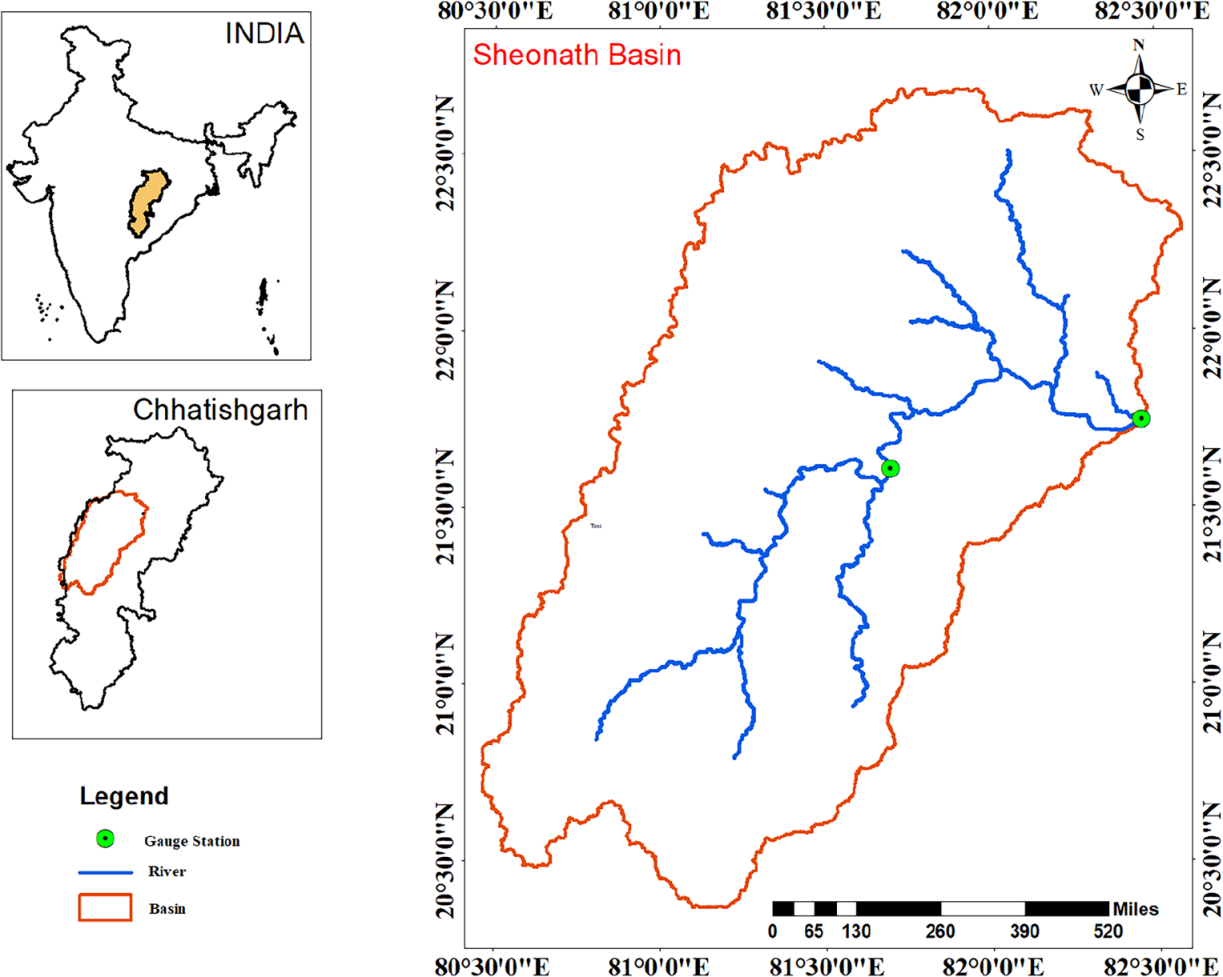
Map of Sheonath basin.

The Sheonath River involves in the Rajnandgaon district, Chhattisgarh, India. The basin is bounded by latitude 20°15ʹ N to 22° 02ʹ N and longitude 80° 26ʹ E to 81° 36ʹ E. The total catchment area is about 30,858 km^2^ (Fig. 1). The length of the river is approximate 379 km. The small tributaries including Arpa, Agrar, Tandula, Kharun, and Hump are associated with the mainstream of Sheonath river. The basin is situated in a tropical climate region. The southwest monsoon is responsible for most of the precipitation in the region. It starts in June and ends on October. Winter or cold season begins from November to February, and the lowest temperature can be found in January. Summer season starts from March to June, and the highest temperatures are measured in the last week of May and June months. The mean rainfall of Sheonath basin is around 1298.60 mm, 51.10 mm, 1132.40 mm, 75.40 mm, and 56.50 mm during the annual, pre-monsoon season, monsoon season, post-monsoon season and winter season, respectively. The area of land use is covered by forest (18.44%), agriculture (72.66%), urban area (2.94%), water (2.04), and barren land (3.92%).

### Data collection

The daily hydrological data (i.e., streamflow and suspended sediment concentration) from 2010–2015 is gathered from the Central Water Commission (CWC), India. Simga and Jondhara stations are located in Sheonath basin. Simga station is situated at 21°37′37″N and 81°41′30″E in Raipur district. Jondhara station is an outlet of Sheonath basin, which is located at 21°42′47″N and 82°21′30″E in Bilaspur district. The suspended sediment concentration samples are collected using the observation of discharge every morning on 08:00 am. While, suspended sediment concentration samples are collected at 0.6 m depth of discharge where the velocity of discharge is measured. The observed data was obtained from a gauging station which was equipped with modern technology.

### Machine learning models

In this study, two of the most common neural network/machine learning structures were chosen for modeling: the cascade correlation neural network (CCNN) and feedforward neural network (FFNN). Moreover, several pair of input combination were also used for forecasting daily suspended sediment concentration in Sheonath basin, India.

#### Cascade correlation neural network (CCNN)

**Figure 2:**
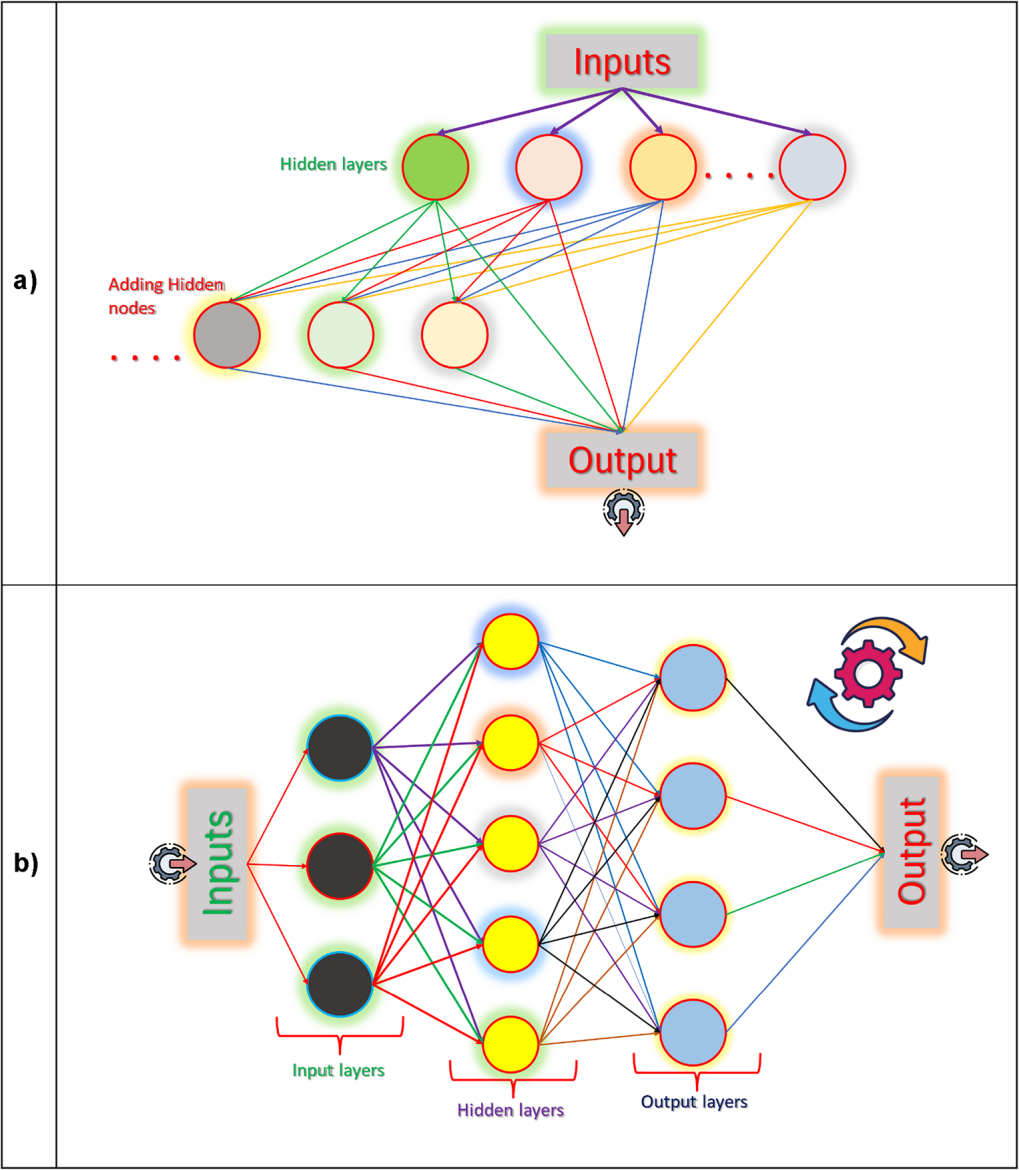
Structure of the models: (**a**) Network of cascade correlation neural network (CCNN) and (**b**) feedforward neural network (FFNN).

A cascade correlation neural network comprises a cascade network, where hidden neurons are added to the hidden layer and do not change after they have been included^65,72,73^. It is known as a cascade because the provision from all neuron’s feeds into new neurons (Fig. 2a).

As new neurons are included to the hidden layer, the learning process expands the extent of connection between the new neurons, and the leftover error of system limits. The goal of this expands the extent of connection between the new neurons OU, the added total output units zero of the correlation degree between value of candidate units (U) and O_o_, the output error (O_e_) observed at unit zero. We describe OU as:

1 $${\text{OU}} = \sum\nolimits_{{\text{o}}} {\left| {\sum\nolimits_{{\text{v}}} {\left( {{\text{U}}_{{\text{v}}} - {\overline{\text{U}}}} \right)\left( {{\text{O}}_{{\text{e,o}}} - {\overline{\text{O}}}_{{\text{o}}} } \right)} } \right|}$$

where O = system output at which the inaccuracy is observed; e = the calibration array. Also, the amounts ${\overline{\text{U}}}$ and ${\overline{\text{O}}}_{{\text{o}}}$ are total mean arrays corresponding to the values of U and O_o_.

To maximize OU, we must compute $\frac{{\partial {\text{OU}}}}{{\partial {\text{w}}_{{\text{i}}} }}$, the fractional derived of OU with regard to candidate unit's received weights (Wi). We can develop and separate the equation for OU to find.

2 $$\frac{{\partial {\text{OU}}}}{{\partial {\text{w}}_{{\text{i}}} }} = \varphi_{{\text{o}}} \sum\nolimits_{{\text{e,o}}} {\left( {{\text{O}}_{{\text{e,o}}} - {\overline{\text{O}}}_{{\text{e}}} } \right){\text{d}}_{{\text{v}}}^{\prime} {\text{I}}_{{\text{i,v}}} }$$

where φ_o_ = the correlated signal between output O and candidate's value, ${\text{d}}_{{\text{v}}}^{\prime}$ = array v of activation functions the candidate units; I_i,v_ = the input the candidate unit collects by I unit for array v.

#### Feedforward neural network (FFNN)

The neural systems, as the name infers, are motivated by their natural partners, the organic mind, and the sensory system. Natural cerebrum is altogether not the same as the customary computerized digital computer as far as its structure and the manner in which it forms data^74,75^. The essential structure of neural systems is a "neuron". A neuron can be seen as a handling unit. In a neural system, neurons are associated with each other through "synaptic weight"s, or "weight"s in short. Every neuron in a system gets "weighted" data by means of these synaptic associations from the neurons that it is associated with and produces a yield bypassing the weighted total of those input signals (either outside contributions from nature or the yields of other neurons) by an “activation function".

In the feedforward system, the hubs in the info (Input) layer get the information signals which are passed to the covered (hidden) layer and afterward to the yield layer (Fig. 2b). The signs are duplicated by the present estimations of loads, and afterward, the weighted information sources are added to yield the net contribution to every neuron of the following layer. The net contribution of a neuron is gone through an enactment or move capacity to deliver the yield of the neuron. The large numbers of literature are already published, therefore we cited only some literatures^75–84^.

### Statistical metrics for performance evaluation

The present study attempts to estimate of suspended sediment concentration by using a variety of discharge and suspended sediment concentration for different time lags variables as inputs to conduct the study. To evaluate the developed models, four distinct statistical performance metrics have been utilized in this study to assess their performance, error, and accuracy ability of predictive models. These three indices are: root mean squared error (RMSE), Nash–Sutcliffe Efficiency Coefficient (N_ES_), Willmott’s Index (WI), and the Legates–McCabe’s index (LM), calculated as follows:

#### Root Mean Square Error (RMSE)

RMSE is a measure of the average magnitude of the errors between predicted and observed values. It calculates the square root of the average squared differences between predicted and observed values over the entire dataset.

3 $${\text{RMSE}} = \sqrt {\frac{{\sum\nolimits_{{{\text{i}} = 1}}^{{\text{n}}} {\left( {{\text{SC}}_{{{\text{Obs}}}} - {\text{SC}}_{{{\text{Pre}}}} } \right)^{2} } }}{{\text{n}}}}$$

where SC_Obs_ is observed sediment concentration, SC_Pre_ is predicted sediment concentration, n is number of observations.

RMSE values range from zero to infinity. RMSE provides insight into the overall model accuracy, with lower values indicating better performance. However, RMSE does not distinguish between systematic and random errors^85–87^.

#### Nash and Sutcliffe Efficiency Coefficient (N_ES_)

N_ES_ is a widely used metric for assessing the predictive accuracy of hydrological and environmental models. It compares the observed data to the model predictions and evaluates how well the model captures the variability of the observed data. It can be calculate using the following formula^88^:

4 $${\text{N}}_{{{\text{ES}}}} = 1 - \frac{{\sum\nolimits_{{{\text{i}} = 1}}^{{\text{n}}} {\left( {{\text{SC}}_{{{\text{Obs}}}} - {\text{SC}}_{{{\text{Pre}}}} } \right)^{2} } }}{{\sum\nolimits_{{{\text{i}} = 1}}^{{\text{n}}} {\left( {{\text{SC}}_{{{\text{Obs}}}} - \overline{{{\text{SC}}}}_{{{\text{Obs}}}} } \right)^{2} } }}$$

where SC_Obs_ is observed sediment concentration, SC_Pre_ is predicted sediment concentration, n is number of observations, $\overline{{{\text{SC}}}}_{{{\text{Obs}}}}$ is average of the observed sediment concentration and ${\overline{{\text{SC}}} }_{{\text{Pre}}}$ is average of predicted sediment concentration.

N_ES_ values range from negative infinity to 1, where a value of 1 indicates perfect agreement between the observed and predicted values, while values closer to 0 indicate poorer performance. N_ES_ is sensitive to errors in both magnitude and timing, making it a comprehensive measure of model performance^86,89,90^.

#### Willmott’s Index of Agreement (WI)

WI is a metric that evaluates the similarity between observed and predicted values relative to the range of variability in the observed data. WI is particularly useful for comparing models across different datasets and variable ranges. It can be calculate using the following formula^90,91^:

5 $${\text{WI}} = 1 - \frac{{\sum\nolimits_{{{\text{i}} = 1}}^{{\text{n}}} {\left( {{\text{SC}}_{{{\text{Obs}}}} - {\text{SC}}_{{{\text{Pre}}}} } \right)^{2} } }}{{\sum\nolimits_{{{\text{i}} = 1}}^{{\text{n}}} {\left( {\left| {{\text{SC}}_{{{\text{Pre}}}} - \overline{{{\text{SC}}}}_{{{\text{Obs}}}} } \right| + \left| {{\text{SC}}_{{{\text{Obs}}}} - \overline{{{\text{SC}}}}_{{{\text{Obs}}}} } \right|} \right)^{2} } }}$$

where SC_Obs_ is observed sediment concentration, SC_Pre_ is predicted sediment concentration, n is number of observations, $\overline{{{\text{SC}}}}_{{{\text{Obs}}}}$ is average of the observed sediment concentration.

It ranges from 0 to 1, where 1 indicates perfect agreement and 0 indicates no agreement beyond the mean of the observed data. WI considers both systematic and random errors, making it a robust measure of model performance^90,92^.

#### Legates–McCabe’s Index (LM)

LM is another index that assesses the agreement between observed and predicted values. It provides insight into how well the model captures the variability and distribution of the observed data. It can be calculate using the following formula^93^:

6 $${\text{LM}} = 1 - \left\lceil {\frac{{\sum\nolimits_{{{\text{i}} = 1}}^{{\text{n}}} {\left| {{\text{SC}}_{{{\text{Obs}}}} - {\text{SC}}_{{{\text{Pre}}}} } \right|} }}{{\sum\nolimits_{{{\text{i}} = 1}}^{{\text{n}}} {\left| {{\text{SC}}_{{{\text{Obs}}}} - \overline{{{\text{SC}}}}_{{{\text{Obs}}}} } \right|} }}} \right\rceil$$

where SC_Obs_ is observed sediment concentration, SC_Pre_ is predicted sediment concentration, n is number of observations, $\overline{{{\text{SC}}}}_{{{\text{Obs}}}}$ is average of the observed sediment concentration.

It ranges from negative infinity to 1, with 1 indicating perfect agreement and values closer to 0 indicating poorer performance. LM is sensitive to systematic errors but less sensitive to random errors compared to other indices.

The following reference values for N_ES_ and WI statistical indices as: very good (0.75 < N_ES_ ≤ 1.0); good (0.65 < N_ES_ ≤ 0.75); satisfactory (0.50 < N_ES_ ≤ 0.65); acceptable (0.40 < N_ES_ ≤ 0.50), and unsatisfactory (NSE ≤ 0.40) describes how the N_ES_ and WI results were analyzed. Considering that RMSE near to zero, and the N_ES_, WI, and LM values would be expected to be a unity for a perfect estimation model^90,94–96^.

## Model development

Daily discharge and suspended sediment concentration data (11/05/2010–10/31/2015) were divided into training (11/05/2010–11/01/2014) and testing (11/02/2014–10/31/2014) data at both stations. Training data contains 1458 data which are about 80% of total data and testing data involves 364 data which are nearly 20% of whole data and explored using CCNN and FFNN models. Statistical analysis of observed data for training and testing phases has been carried out to determine the behaviour of data characteristics using mean, minimum, maximum, median, standard deviation, skewness, and kurtosis as given in Table 1.

**Table 1:** The daily statistical parameters of sediment and discharge data sets.

Station	Dataset	Data type	No of data	Minimum	Maximum	Median	Mean	Std deviation	Skewness	Kurtosis
Jondhara	Training	Sediment concentration (mg/L)	1458	0.00	988.00	0.00	80.19	168.90	2.20	4.07
		Discharge (m^3^/s)	1458	0.00	9193.26	0.00	284.51	703.93	4.76	37.52
	Testing	Sediment concentration (mg/L)	364	0.00	1220.00	0.00	82.79	168.92	3.04	11.67
		Discharge (m^3^/s)	364	0.00	6528.58	0.00	547.43	994.81	2.71	9.67
Simga	Training	Sediment concentration (mg/L)	1458	0.00	890.00	1.00	39.39	90.48	3.48	16.04
		Discharge (m^3^/s)	1458	0.00	7358.73	7.71	144.13	462.15	7.42	79.02
	Testing	Sediment concentration (mg/L)	364	0.00	863.00	0.00	73.58	116.07	1.99	6.18
		Discharge (m^3^/s)	364	0.00	6844.26	7.37	293.04	735.65	6.02	45.44

The minimum values showed that there was a period when no discharge and suspended sediment concentration condition prevailed, while maximum values provided discharge and suspended sediment concentration values was fluctuating considerably during training and testing phases at Jondhara station (i.e., maximum discharge (training phase) and maximum suspended sediment concentration (testing phase) and Simga station (i.e., maximum discharge and suspended sediment concentration (testing phase). Median values of discharge and suspended sediment concentration at Jondhara station were found zero which shows that half of the data are zero. Although, at Simga station, the median values are found as non-zero positive value (e.g., except for suspended sediment concentration during testing phase). Maximum and minimum mean values of discharge and suspended sediment concentration were found during training (Simga station) and testing phases (Jondhara station), respectively. Also, the values of standard deviation were calculated at both stations, and highest deviation in discharge was found during testing phase (Jondhara station), while highest deviation in suspended sediment concentration was found during testing phase (Jondhara station).

Also, the highest skewness in discharge was found during training phase at Simga station, while Jondhara station during training phase showed the highest skewness in suspended sediment concentration. Based on the statistical survey for discharge and suspended sediment concentration, it can be found that applying data is highly fluctuating and is not normally distributed.

Different combinations of lag-times discharge *Q*_*t-n*_ (i.e., 1- and 2-days) and suspended sediment concentration *S*_*t-n*_ (i.e., 1-, 2-, 3, and 4-days) as input variables and current suspended sediment concentration (S_t_) as the desired variable were investigated to develop CCNN and FFNN models. Therefore, nine inputs combinations were developed based on correlation of lag-times discharge and suspended sediment concentration which is presented in Table 2.

**Table 2:** Input combinations.

No	Input combination	Output	FFNN	CCNN
1	S_t-1_	S_t_	FFNN1	CCNN1
2	S_t-1_, S_t-2_	S_t_	FFNN2	CCNN2
3	S_t-1_, S_t-2_, S_t-3_	S_t_	FFNN3	CCNN3
4	S_t-1_, S_t-2_, S_t-3_, S_t-4_	S_t_	FFNN4	CCNN4
5	S_t-1_, Q_t-1_	S_t_	FFNN5	CCNN5
6	S_t-1_, S_t-2_, Q_t-1_	S_t_	FFNN6	CCNN6
7	S_t-1_, Q_t-1_, Q_t-2_	S_t_	FFNN7	CCNN7
8	S_t-1_, S_t-2_, Q_t-1_, Q_t-2_	S_t_	FFNN8	CCNN8
9	S_t-1_, S_t-2_, S_t-3_, Q_t-1_, Q_t-2_	S_t_	FFNN9	CCNN9

First four combinations (i.e., combinations 1–4) were developed based on only lag-times suspended sediment concentration ta while other combinations (i.e., combinations 5–9) developed using both discharge and suspended sediment concentration data.

**Figure 3:**
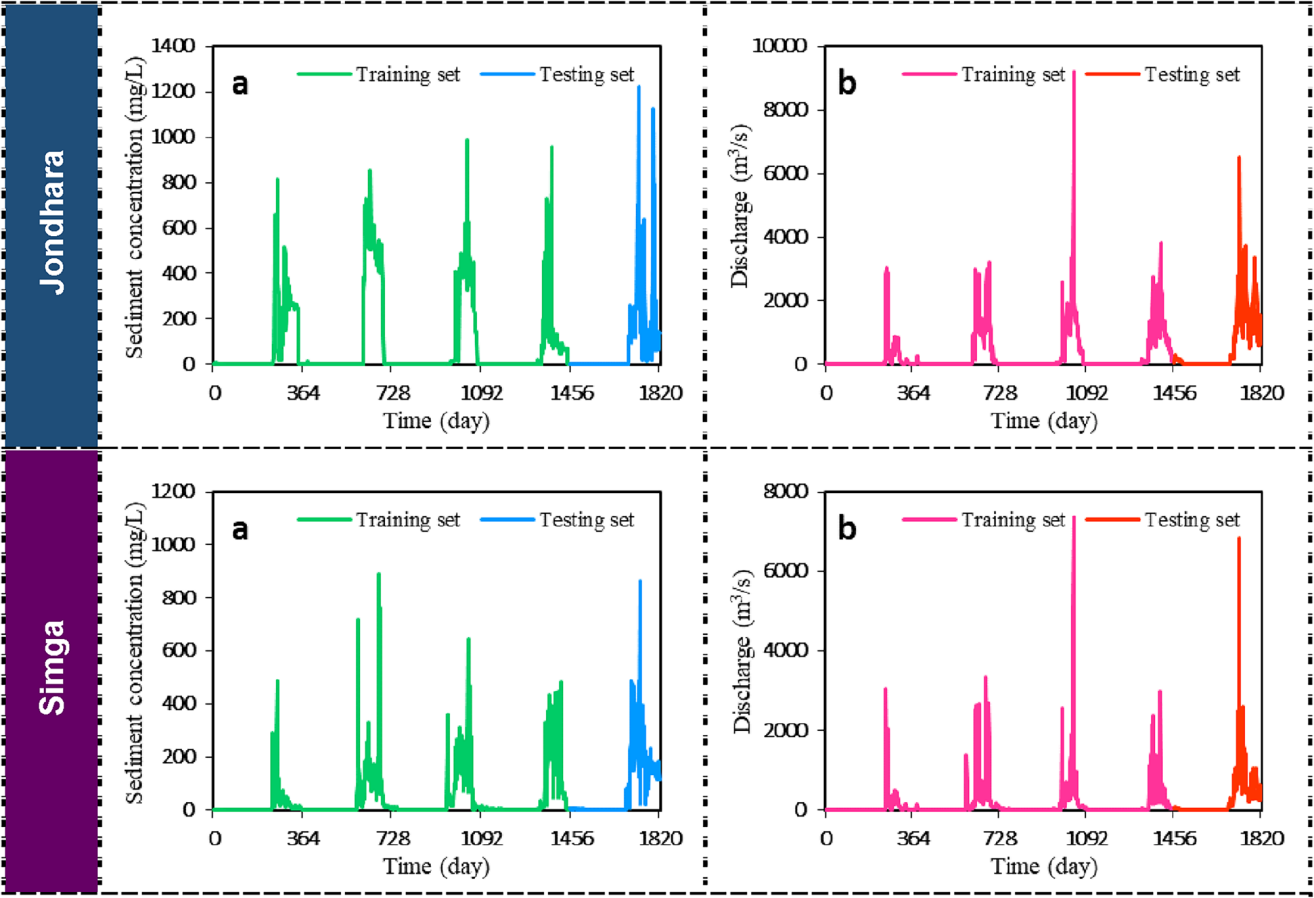
Time series plot for the data period of (2010/11/05–2015/10/31): (**a**) sediment; (**b**) discharge.

Daily discharge and suspended sediment concentration data are plotted separately with time scale (training and testing phases) on X-axis and corresponding discharge or suspended sediment concentration on Y-axis at both stations. Time series plotting of suspended sediment concentration at Jondhara station clearly showed there was considerable suspended sediment concentration with maximum suspended sediment concentration in monsoon period (testing phase). However, the rest year were found as negligible suspended sediment concentration in Fig. 3. Also, at Jondhara station, the discharge was found only in monsoon period with maximum discharge (training phase). At Simga station, the peak discharge and suspended sediment concentration were found during testing phase. Figure 3 illustrates that many values in the dataset were zero due to the non-perennial nature of the river, where continuous water flow was absent during certain periods. Consequently, sediment concentrations were also zero during these periods. This resulted in a mix of zero values and non-zero data in the dataset. The presence of zero values is further attributed to the validation data, which also captured periods of zero stream flow.

## Results

### Quantitative assessment of developed models based on statistical indices

All the input combinations were investigated using CCNN and FFNN models with one hidden layer and different numbers of neurons in hidden layer. FFNN models with selected input combinations were developed for determining a single hidden layer at both stations. The number of neurons was increased to improve the model performance. When the model performance was not improved by adding the neurons, the neurons were not added in the hidden layer. The best model was determined based on the results of performance during testing phase. The model with a minimum value of RMSE and maximum values of N_ES_, WI and LM was selected as the best model and given in Table 3 at both stations.

**Table 3:** The result of FFNN model for different input combinations of Jondhara and Simga.

Station	Model	Model structure	Training data				Testing data			
			RMSE (mg/L)	N_ES_	WI	LM	RMSE (mg/L)	N_ES_	WI	LM
Jondhara	FFNN1	1-8-1	53.49	0.900	0.973	0.878	112.95	0.552	0.859	0.649
	FFNN2	2-2-1	54.36	0.896	0.972	0.859	113.68	0.546	0.847	0.635
	FFNN3	3-5-1	54.08	0.897	0.972	0.877	108.33	0.588	0.861	0.663
	FFNN4	4-13-1	59.93	0.874	0.965	0.813	108.15	0.589	0.863	0.598
	FFNN5	2-11-1	56.58	0.888	0.969	0.846	114.28	0.541	0.872	0.576
	FFNN6	3-8-1	58.68	0.879	0.966	0.722	110.80	0.569	0.860	0.441
	FFNN7	3-15-1	56.45	0.888	0.969	0.812	109.28	0.580	0.867	0.573
	**FFNN8**	**4-6-1**	**58.77**	**0.879**	**0.966**	**0.822**	**107.86**	**0.591**	**0.867**	**0.588**
	FFNN9	5-12-1	53.73	0.899	0.973	0.867	109.77	0.577	0.886	0.624
Simga	FFNN1	1-10-1	49.54	0.700	0.902	0.715	57.09	0.757	0.924	0.775
	FFNN2	2-10-1	49.11	0.705	0.907	0.728	54.23	0.781	0.932	0.794
	**FFNN3**	**3-12-1**	**48.58**	**0.712**	**0.910**	**0.741**	**54.04**	**0.783**	**0.936**	**0.780**
	FFNN4	4-19-1	49.05	0.706	0.910	0.728	55.23	0.773	0.934	0.777
	FFNN5	2-10-1	48.23	0.716	0.907	0.644	56.24	0.765	0.928	0.734
	FFNN6	3-14-1	47.47	0.725	0.913	0.709	55.00	0.775	0.933	0.760
	FFNN7	3-5-1	46.61	0.734	0.916	0.719	56.32	0.764	0.930	0.764
	FFNN8	4-4-1	48.57	0.712	0.907	0.722	55.09	0.774	0.930	0.791
	FFNN9	5-6-1	47.07	0.729	0.915	0.743	55.40	0.772	0.932	0.776

Bold values show the best model structure with lowest error and highest model efficiency.

All eighteen developed FFNN models developed precise daily suspended sediment concentration estimations during training with a range of RMSE (mg/L), N_ES_, WI and LM between 46.610 to 59.930 (mean = 52.239) (mg/L), 0.700 to 0.900 (mean = 0.802), 0.902 to 0.973 (mean = 0.940) and 0.644 to 0.878 (mean 0.775) respectively, and for testing with a range of 54.040 to 114.280 (mean = 82.986) (mg/L), 0.541 to 0.783 (mean = 0.671), 0.847 to 0.936 (mean = 0.898) and 0.441 to 0.794 (mean = 0.683). Based on the values of N_ES_ for the Jondhara station were found satisfactory satisfactory (0.50 < N_ES_ ≤ 0.65) while for the Simga station N_ES_ were found good as 0.75 < N_ES_; and WI for both stations were higher than 0.75, showing high precision in predicting daily suspended sediment concentration.

FFNN8 model with four inputs and six neurons in the hidden layer (4-6-1 structure) at Jondhara station was carefully determined as the best on after comparing the performance of all models given in Table 3. For training phase, the values of RMSE, N_ES_, WI, and LM were found as 58.77 mg/L, 0.879, 0.966, and 0.966, respectively, while for testing phase, the values provided as 107.86 mg/L, 0.591, 0.867, and 0.588, respectively, for the selected FFNN8 model. While, FFNN5 model performed worst with RMSE, N_ES_, WI and LM values of 56.58 mg/L, 0.888, 0.969, 0.846 during training phase and 114.28 mg/L, 0.541, 0.872, and 0.576 during testing phase of the developed models. After comparison, FFNN3 model was chosen as the best model at Simga station with the values of RMSE, N_ES_, WI, and LM as 48.58 mg/L, 0.712, 0.910, and 0.741 during training phase and 54.04 mg/L, 0.783, 0.936, and 0.780, during the testing phase, respectively. While, FFNN1 model performed worst with RMSE, N_ES_, WI and LM values of 49.54 mg/L, 0.700, 0.902, 0.715 during training phase and 57.09 mg/L, 0.757, 0.924, and 0.775 during testing phase of the developed models. The training performance of the best model was better compared to testing phase.

Quantitative evaluation of CCNN model is given in Table 4. Selected input combinations as given in Table 1 were used for CCNN model development; model with single hidden layer and different input and hidden neurons. To get a better performance among individual CCNN models, a comparison has been made to obtain the best CCNN model at both stations. All eighteen developed CCNN models developed precise daily suspended sediment concentration estimations during training with a range of RMSE (mg/L), N_ES_, WI and LM between 46.350 to 58.410 (mean = 52.383) (mg/L), 0.692 to 0.895 (mean = 0.798), 0.900 to 0.971 (mean = 0.9380) and 0.692 to 0.861 (mean 0.781) respectively, and for testing with a range of 53.710 to 108.990 (mean = 80.361) (mg/L), 0.582 to 0.785 (mean = 0.690), 0.866 to 0.937 (mean = 0.904) and 0.618 to 0.788 (mean = 0.713). Based on the testing data values of N_ES_ for the Jondhara station were found satisfactory (0.50 < N_ES_ ≤ 0.65) while for the Simga station N_ES_ were found good as 0.75 < N_ES_; and WI for both stations were higher than 0.75, showing high precision in predicting daily suspended sediment concentration.

**Table 4:** The result of CCNN model for different input combinations of Jondhara and Simga.

Station	Model	Model structure	Training data				Testing data			
			RMSE (mg/L)	N_ES_	WI	LM	RMSE (mg/L)	N_ES_	WI	LM
Jondhara	CCNN1	1-5-1	56.40	0.888	0.969	0.861	107.18	0.596	0.868	0.669
	CCNN2	2-3-1	56.48	0.888	0.969	0.855	108.14	0.589	0.866	0.658
	CCNN3	3-1-1	57.32	0.885	0.968	0.850	107.57	0.593	0.869	0.647
	**CCNN4**	**4-2-1**	**57.02**	**0.886**	**0.969**	**0.845**	**98.02**	**0.662**	**0.890**	**0.668**
	CCNN5	2-6-1	54.84	0.895	0.971	0.859	99.62	0.651	0.889	0.657
	CCNN6	3-5-1	56.88	0.887	0.968	0.849	99.47	0.652	0.893	0.669
	CCNN7	3-2-1	58.18	0.881	0.966	0.850	108.67	0.585	0.872	0.647
	CCNN8	4-2-1	58.41	0.880	0.967	0.844	105.67	0.608	0.876	0.642
	CCNN9	5-0-1	57.97	0.882	0.967	0.836	108.99	0.582	0.866	0.618
Simga	CCNN1	1-5-1	50.21	0.692	0.900	0.695	57.07	0.758	0.926	0.781
	CCNN2	2-4-1	48.84	0.709	0.907	0.723	54.96	0.775	0.933	0.783
	CCNN3	3-5-1	47.91	0.719	0.911	0.732	54.68	0.777	0.933	0.776
	**CCNN4**	**4-3-1**	**48.82**	**0.709**	**0.908**	**0.722**	**53.71**	**0.785**	**0.936**	**0.788**
	CCNN5	2-4-1	48.44	0.713	0.908	0.692	58.20	0.748	0.925	0.747
	CCNN6	3-2-1	49.81	0.697	0.902	0.709	56.15	0.765	0.929	0.773
	CCNN7	3-4-1	46.35	0.737	0.916	0.724	58.45	0.746	0.926	0.747
	CCNN8	4-2-1	49.52	0.700	0.904	0.697	56.03	0.766	0.929	0.779
	CCNN9	5-3-1	47.68	0.722	0.911	0.722	53.91	0.784	0.937	0.778

Bold values show the best model structure with lowest error and highest model efficiency.

CCNN4 model was selected as the best model for forecasting suspended sediment concentration at Jondhra station, and the values of RMSE, N_ES_, WI, and LM as 57.02 mg/L, 0886, 0.969 and 0.845, respectively during training phase and 95.02 mg/L, 0.662, 0.890, and 0.668, respectively during testing phase. The CCNN9 model performed worst for Jondhra station. For Sigma station, CCNN4 model with four input variables and three neurons in hidden layer was selected as the best model among individual CCNN models. The values of RMSE, N_ES_, WI, and LM were found 48.82 mg/L, 0.709, 0.908, and 0.722, respectively during training phase and 53.71 mg/L, 0.785, 0.936, and 0.788, respectively during testing phase. For Simga station, CCNN7 model performed worst compared to all other developed models.

Comparison between the best models (i.e., FFNN8 and CCNN4) at Jondhara station explained that CCNN4 model performed better compared to FFNN8 model during testing phase clearly. Therefore, CCNN4 model was selected as the best model for forecasting suspended sediment concentration at Jondhara station. In addition, comparison between the best models (i.e., FFNN3 and CCNN4) at Simga station revealed that CCNN4 model was a little accurate compared to FFNN3 model during testing phase. CCNN4 model, therefore, was determined as the best model for forecasting suspended sediment concentration at Simga station.

### Qualitative assessment of developed models based on visual interpretation

**Figure 4:**
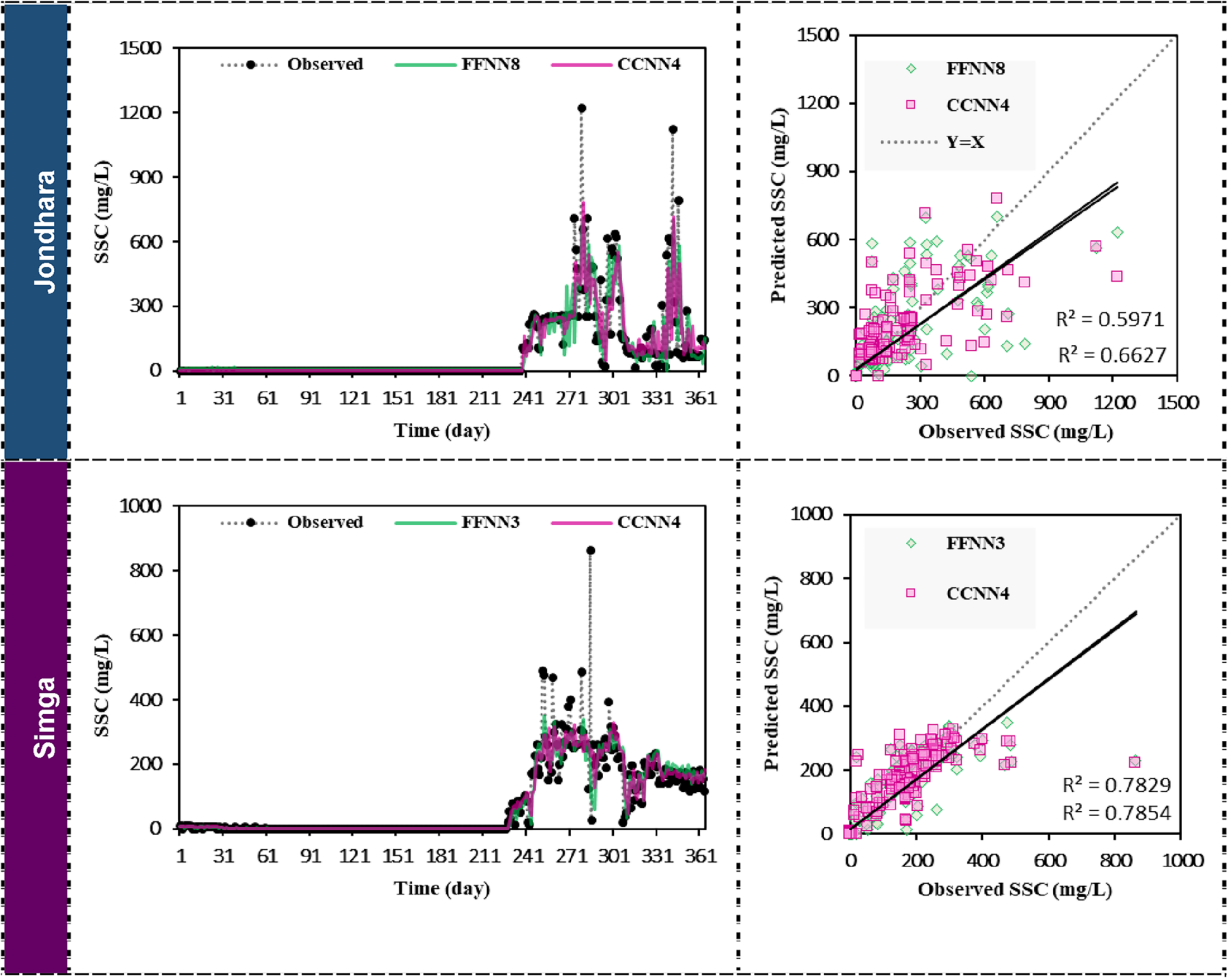
Forecasted suspended sediment concentration (SSC) by the best FFNN and CCNN models (left side) and scatter diagrams (right side) at Jondhara and Simga stations in testing phase.

Qualitative evaluation of selected models was carried out based on the line diagram and scatter plots as depicted in Fig. 4. The forecasted suspended sediment concentration using FFNN8 and CCNN4 models during testing phase was plotted compared to observed suspended sediment concentration at Jondhara station. The forecasted values at both stations showed the superior agreement compared to observed ones during low observed data. However, when the values of suspended sediment concentration increase, the clear fluctuation in the forecasted values was provided. In addition to fluctuation, FFNN8 and CCNN4 model gave the under-forecasted values compared to peak observed ones. The relationship between forecasted values between FFNN8 and CCNN4 models was judged using the scatter plot. From the plotting, it could be seen that there was a good agreement between FFNN8 and CCNN4 models.

Line diagram at Simga station provided accurate agreement until 215 days. After that, the values of forecasted suspended sediment concentration for FFNN3 and CCNN4 models were fluctuated with under-forecasted values compared to the peak ones (Fig. 4). While the very good agreement was found between FFNN3 and CCNN4 models at Simga station, the agreement is better compared to Jondhara station.

**Figure 5:**
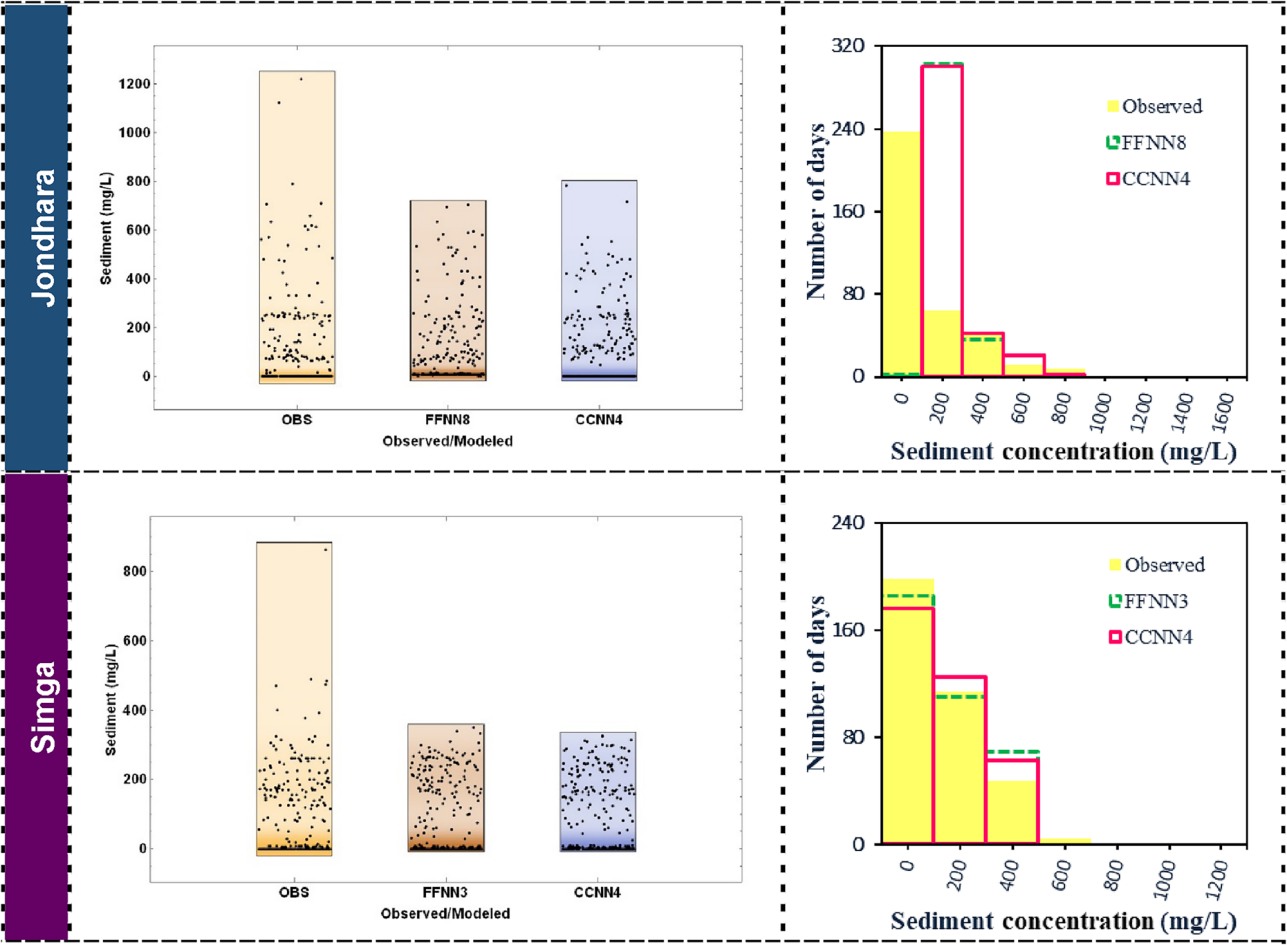
Density plots (left side) and histograms (right side) of observed and the best FFNN and CCNN models for testing phase at Jondhara and Simga stations.

Distribution of discharge and suspended sediment concentration on time was carried out using density plots and histograms as represented in Fig. 5 Observed and forecasted values using FFNN and CCNN models were plotted in density plots and histograms. From the density plot and histogram based on Jondhara station, the observed values less than 150 mg/L were found as maximum data, while FFNN8 and CCNN4 models provided the maximum data between 100 and 300 mg/L. In case of Simga station, the similarity in the data distribution between observed and forecasted suspended sediment concentration using FFNN3 and CCNN4 models was found as shown in Fig. 5. The maximum data point of all three sets were found among values 0–100 mg/L, 100–300 mg/L, and 300–500 mg/L. Since the similarity was outstanding at Simga station, the best models (i.e., FFNN3 and CCNN4) forecasted suspended sediment concentration accurately.

**Figure 6:**
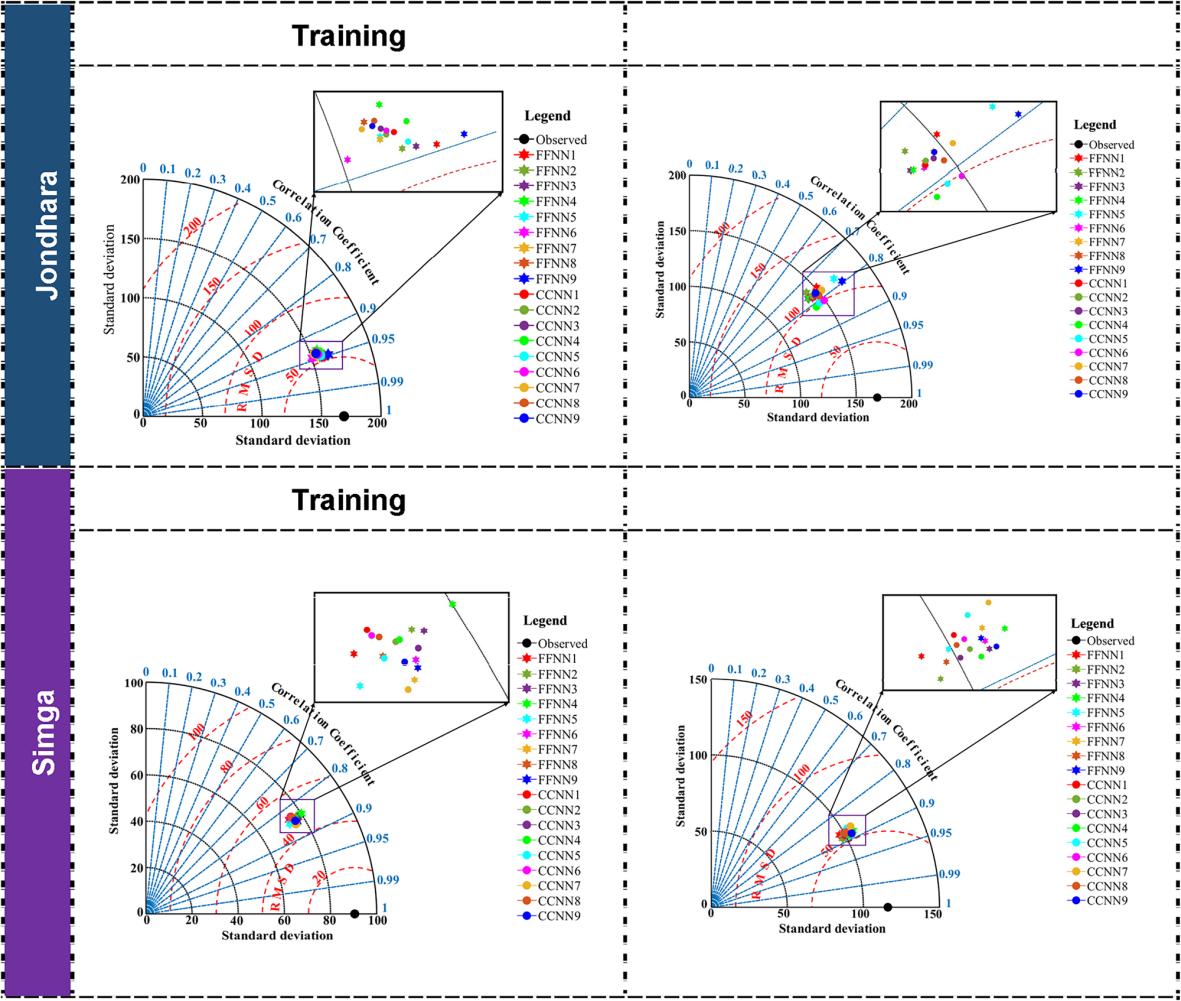
Taylor diagram of the models in the training and testing phase for best model of each model type at Jondhara and Simga stations.

Taylor diagram is a single-window for comparing the performance of different models based on three different statistical indices, including Pearson’s correlation coefficient (R), RMSE, and standard deviation (SD)^97–101^. Taylor diagram, therefore, was applied to assess performance of FFNN and CCNN models based on observed suspended sediment concentration graphically using CC, RMSE, and SD during training and testing phases at both stations. For all the models at Jondhara station, the value of CC was found nearly 0.95, the value of SD was provided below 200, and the value of RMSE was put below 100 mg/L during training phase as given in Fig. 6, while the value of CC was found above 0.7, the value of SD was provided near to 150 except for FFNN5 and FFNN9, and the value of RMSE was put below 150 mg/l.

In case of Simga station, the value of CC was showed between 0.80 and 0.90 for all models, the value of SD was produced between 50 and 100, and the value of RMSE was yielded nearly 50 mg/l for all the models during the training period as depicted in Fig. 6. Although the value of CC was showed slightly less than 0.9 for all the models, the value of SD was produced nearly 100, and the values of RMSE was yielded slightly greater than 50 mg/L for all the models. After analysing Taylor diagram of different models, it is not clear that which model is the best model. After comparing the results of different developed models based on RMSE, N_ES_, WI, and LM values, it can be concluded that the potential of CCNN model for forecasting suspended sediment concentration was better compared to FFNN model at both stations. Based on the visual comparisons (i.e., line diagram, scatter diagram, density plot, histograms, and Taylor diagram) during testing phase, CCNN model was more accurate based on density plot and histograms. In addition, the scatter diagram indicated that CCNN model showed less deviation from Y = X line compared to CCNN model for forecasting suspended sediment concentration during testing phase at both stations. In finding of the best models, all models indicated the different results for forecasting suspended sediment concentration at both stations. It was also found that the model parameters can be considered as the main factors to find the best input arrangements. It can be concluded that that CCNN and FFNN models could be forecasted suspended sediment concentration within satisfactory and accurate category.

## Discussion

The performance of the FFNN and CCNN based models was influenced by the choice of the input considered for both the stations. The best performance of the FFNN model was achieved when one day and two days lagged values of suspended sediment concentration and discharge were considered for Jondhara station. The model structure corresponds for the best suspended sediment model at Jondhara station was 4-6-1 (Input-hidden layer and output layer)). While, for the Simga station, the best model was FFNN3 having inputs of one day, two days and three days legged values of suspended sediment load. The addition of discharge data as input variables had reduced the performance of the FFNN model for Simga station. This model structure of the FFNN3 was 3-12-1. The comparative performance of the models FFNN at two stations for predicting suspended sediment load showed the inputs plays a key role in predicting capability of the model. The effects of the ANN architecture greatly influenced the models performance and this funding is align with Shukla et al.^102^ and Rajput et al.^103^. Essem et al.^57^ compared SVM, ANN, and LSTM to forecast suspended sediment load in Malaysia. Among these models, the ANN3 model, formulated using the ANN algorithm and input scenario 3 (comprising current-day sediment flow, previous-day sediment flow, and previous-day suspended sediment load), emerged as the most effective model for prediction. Our findings using the FFNN model are in line with the results reported by Essem et al.^57^. The efficacy of the models in predicting suspended sediment load is better with multiple inputs as compared to single input variable.

The best performance of the CCNN model was achieved when one day, two days, three days and four days lagged values of suspended sediment load were considered for Jondhara station. The model structure corresponds for the best suspended sediment model at Jondhara station was 4-2-1 (Input-hidden layer and output layer)). While, for the Simga station, the best model was CCNN4 having inputs of one day, two days and three days and four days lagged values of suspended sediment load. The addition of discharge data as input variables had reduced the performance of the CCNN model for both the stations. This model structure of the CCNN4 was 4-3-1. The comparative performance of the models CCNN at two stations for predicting suspended sediment load showed the inputs plays a key role in predicting capability of the model. Our study findings using the CCNN model disagreed with the Jimeno-Sáez^104^ which reported that the performance of the machine learning models found best considering all the inputs. However, we found that the performance of the CCNN reduced while adding the discharge data with the suspended sediment load. Elbisy et al.^105^ compared feed-forward back propagation neural network (FFNN) and cascade correlation neural network (CCNN), and found CCNN model produces slightly more accurate results, and it also learns almost as fast as the BP model when compared to the FFNN model. The present study finding align with this finding.

Recent study also showed the accuracy and effectiveness of using a different input with different architecture for removing error in the time series data and, thus, enhancement of model forecasting accuracy in assessment to a standalone model^38,47,106–115^. Overall, our study demonstrates the model structure (input-hidden-output) layers, the suspended sediment load carrying the stream/river and the algorithm used influences the predicting capability of the models. The FFNN model showed better performance considering the discharge flow data along with the suspended sediment load data, however, CCNN model showed optimum performance with suspended sediment load data alone. As seen from the result, it is clear that for both the stations, different models with different architectures, giving different results. But if what comparison is to be made between the two models, then CCNN4 models for both the stations are giving more accurate results. The CCNN4 model is capable of giving better results by understanding the hydrological complexity well. The quantification of the suspended sediment load is essential for planning of desilting of the reservoirs, water availability assessment and ascertaining the capacity of the reservoirs. Our study results could play significant role in accurate prediction of the suspended sediment load and the developed methodology may be evaluated at other places for its accuracy.

## Shortcomings and future study

Accurate forecasting of suspended sediment concentration (SSC) is crucial tasks for water resource management, flood prediction, erosion control and ecosystem conservation. The proposed input combination to compare Cascade Correlation Neural Network (CCNN) and Feedforward Neural Network (FFNN) model offers enhanced precision and reliability in predicting SSC, facilitating informed decision-making for policymakers and stakeholders. Additionally, integrating hybrid algorithms can notably boost the predictive accuracy of suspended sediment concentration models. Therefore, upcoming research should explore the integration of hybrid models to improve prediction accuracy further. Ultimately, this research underscores the promise of CCNN and FFNN approaches in suspended sediment concentration prediction with small data sets, emphasizing the importance of continued exploration in this field.

Employing model ensemble techniques, such as combining predictions from multiple machine learning models or integrating machine learning with physical-based models, can potentially improve predictive accuracy and reliability by leveraging the strengths of different modeling approaches.

## Conclusions

In the current study, CCNN and FFNN models were used to forecast daily suspended sediment concentration at Jondhara and Sigma stations, India. The suspended ssediment concentration forecasting was carried out for both stations with nine input combinations which contained the previous one- and two-day discharge and one-, two-, three-, and four-day suspended sediment concentration. The total data was divided into training data and testing data. The performance of developed models was examined using statistical indices based on RMSE, N_ES_, WI, and LM values. The model has the lowest value of RMSE and is close to zero and the highest value and is close to one of N_ES_, WI, and LM values, were the best-chosen the best input combination model. Based on quantitative and visual observation, FFNN8 model at Jondhara station and FFNN3 model at Simga station were found the best models among different model architectures explored in FFNN technique. The values of RMSE, N_ES_, WI, and LM during the training and testing phases indicated that FFNN8 with input (*S*_*t-1*_, *S*_*t-2*_*, Q*_*t-1*_*, Q*_*t-2*_) and FFNN3 (*S*_*t-1*_, *S*_*t-2*_*, S*_*t-3*_) models have the best performance out of nine FFNN models at both stations. The architectures 4-2-1 and 4-3-1 of CCNN model with input (*S*_*t-1*_, *S*_*t-2*_*, S*_*t-3*_*, S*_*t-4*_) combination were considered as the best models for forecasting suspended sediment concentration at both stations. Owing to the deficiency of overfitting during the training period, the model was selected based on performance during the testing period to select the model with stable results. Based on the comparison of FFNN and CCNN models performance, CCNN model was found to have a good proximity with observed values at Jondhara station, while CCNN4 provided slightly better performance than FFNN3 model for Simga station. After comparing the results of different developed models based on RMSE, N_ES_, WI, and LM values, it can be concluded that the potential of CCNN model for forecasting suspended sediment concentration was better compared to FFNN model algorithm at both stations in Sheonath basin, India. It can be confirmed from the current study that CCNN and FFNN models can be applied to perform better forecasting of hydrological variables with non-linear and complex relationship. Every station has a specific networked model which could model the data more precisely preciously.

The sources of uncertainty in predicting Suspended Sediment Load (SSL) are multifaceted and can stem from various factors, influencing the reliability and accuracy of predictive models. Some of the key sources of uncertainty include: (a) Data Variability (b) Model Complexity (c) Parameter Estimation (d) Input Data Quality and (e) Model Selection. In summary, while both CCNN and FFNN have their strengths and weaknesses, the choice between them depends on the specific requirements of the sediment concentration prediction task, including data complexity, computational resources, and desired prediction accuracy. A comparative analysis can help researchers and practitioners understand the trade-offs between these models and select the most suitable one for their applications.

## Data Availability

The datasets used and/or analyzed during the current study are available from the corresponding author upon reasonable request.

## Abbreviations

SSC: Suspended sediment concentration
CCNN: Cascade correlation neural network
FFNN: Feed Forward neural network
N_ES_: Nash and Sutcliffe efficiency coefficient
RMSE: Root mean square error
WI: Willmott’s index of agreement
LM: Legates–McCabe’s index
SRC: Sediment rating curve
MLR: Multiple linear regression
ML: Machine learning
ANN: Artificial neural network
FFBPNN: Feedforward backpropagation neural network
SVM: Support vector machine
R: Pearson’s correlation coefficient
RSS: Random subspace
RF: Random forest
RBF: Radial basis function kernel
NPK: Normalized polynomial kernel
WANN: Wavelet based artificial neural network
SM-LSTM: Selective multimodal long short-term memory network
LSTM: Long short-term memory network
RNN: Recurrent neural network
MLP: Multilayer perceptron
DO: Dissolved oxygen
TDS: Total dissolved solid
pH: Potential of hydrogen
RBNN: Radial basis neural networks
WGRNN: Wavelet-generalized regression neural network
GRNN: Generalized regression neural network
Km: Kilometer
CWC: Central Water Commission

## Code availability

The codes used during the current study are available from the corresponding author upon reasonable request.
